# Multi-omics mendelian randomization integrating GWAS and eQTL data revealed potential drug target for irritable bowel syndrome

**DOI:** 10.3389/fgene.2026.1798264

**Published:** 2026-05-19

**Authors:** Huiwen Ke, Wenchao Chen, Qian Zhang, Zipeng Liu, Pengfei Wei, Li Li, Haitao Yu, Dongmei Huang, Chaoting Lan, Ning Xu, Lei Pi, Kai Song

**Affiliations:** 1 Department of Laboratory Medicine, The Second Clinical College of Guangzhou University of Chinese Medicine, Guangzhou, China; 2 Department of Pediatrics, The First Affiliated Hospital of Jinan University, Jinan University, Guangzhou, China; 3 Department of Pediatrics, Nanfang Hospital, Southern Medical University, Guangzhou, Guangdong, China; 4 Guangzhou Women and Children’s Medical Center, Guangzhou Medical University, Guangzhou, China; 5 Luoyang Women and Children Hospital, Luoyang, Henan, China; 6 The First School of Clinical Medicine, Southern Medical University, Guangzhou, China

**Keywords:** drug repurposing, eQTL, GWAS, irritable bowel syndrome, mendelian randomization

## Abstract

**Introduction:**

Irritable bowel syndrome (IBS) is a common gastrointestinal disorder mainly affecting the young and female with limited therapeutic options, necessitating the identification of novel drug targets. This study aimed to identify and prioritize new, genetically validated drug targets for IBS by leveraging large-scale human genetic data.

**Methods:**

We conducted a systematic, druggable genome-wide Mendelian randomization (MR) analysis to evaluate the causal effects of 5,642 potential druggable protein-coding genes on IBS risk. The analysis integrated summary statistics from the largest available IBS genome-wide association study (GWAS), including 53,400 cases and 433,201 controls, with comprehensive blood expression quantitative trait loci (eQTL) data. Significant findings were further validated using colocalization analysis. A phenome-wide association study (PheWAS) was performed to assess the potential for on-target adverse effects. Finally, potential therapeutic compounds were predicted using the Drug Signatures Database (DSigDB) and molecular docking.

**Results:**

The MR analysis identified eight genes with potential causal associations with IBS. Following rigorous validation with colocalization analysis, EP300 and P2RY14 emerged as the most promising candidate targets. Genetically predicted higher expression of both EP300 (OR: 1.128, 95% CI: 1.079-1.180) and P2RY14 (OR: 1.118, 95% CI: 1.067-1.172) was suggestively causally associated with an increased risk of IBS. The PheWAS analysis indicated that EP300 and P2RY14 did not show genome-wide significant associations with any other phenotypes. Additionally, molecular docking predicted that existing compounds, such as captopril and menadione, could effectively bind to the EP300 protein.

**Conclusion:**

Our study provides genetic evidence establishing EP300 and P2RY14 as promising drug targets for the treatment of IBS, laying a foundation for future drug development and repurposing efforts.

## 1 Introduction

Irritable bowel syndrome (IBS) is a prevalent chronic functional gastrointestinal disorder. Its primary characteristics include recurrent episodes of abdominal pain or discomfort, occurring in the absence of evidence of organic lesions and biochemical abnormalities, accompanied by alterations in bowel habits (Agrawal and Whorwell, 2006). These manifestations significantly impair patients’ quality of life and daily functioning. The prevalence of IBS varies from 1.1% to 35.5% (Huang et al., 2023) and the disorder predominantly affects females, with an incidence approximately 1.5–2 times higher than that in males. Moreover, IBS is among the commonest functional gastrointestinal disorders (FGIDs) in children (Devanarayana and Rajindrajith, 2018) and the prevalence in Asian children was reported to be of 12.4% (Devanarayana et al., 2015). IBS imposes a substantial disease burden on the healthcare system (Chey et al., 2015), for instance, the annual direct healthcare costs of irritable bowel syndrome in the UK range from £130 million to £2 billion.

Despite its prevalence, the management of IBS remains a clinical challenge. Current treatment strategies are often directed at symptom relief and include dietary modifications, lifestyle changes, and pharmacotherapy such as antispasmodics, antidiarrheals, laxatives, and antidepressants (Agrawal and Whorwell, 2006). However, these treatments offer only modest efficacy, and many patients experience persistent or relapsing symptoms, leading to a high degree of dissatisfaction (Agrawal and Whorwell, 2006; Saha, 2014). The heterogeneity of IBS subtypes and symptom presentations further complicates the development of a “one-size-fits-all” therapy. Consequently, there is an urgent and unmet need to identify novel therapeutic targets to develop more effective and targeted treatments for IBS (Halland and Saito, 2015).

Human genetic research is now widely applied in drug development for a variety of complex diseases. Integrating genetic evidence into drug discovery and development—particularly by prioritizing genetically supported drug targets—represents one of the most effective strategies as such targets are more likely to succeed in clinical trials (Duffy et al., 2024; Abou-Karam et al., 2024). Druggable genes refer to those in the human genome that encode proteins capable of being specifically bound and modulated by therapeutic agents (Sharma et al., 2024). These genes serve as prime candidates for precision medicine and play a pivotal role in targeted therapies, drug repurposing, and the development of novel therapeutic strategies. Genome-wide association studies (GWAS) datasets offer broad prospects for drug repurposing based on genetic discoveries. For instance, among the 50 drugs approved by the U.S. Food and Drug Administration (FDA) in 2021, two-thirds were supported by corresponding genetic evidence, underscoring the considerable potential of GWAS in drug development (Kang et al., 2023). Mendelian randomization (MR) is a powerful genetic epidemiological approach that uses genetic variants as instrumental variables to infer causal relationships between exposures and disease outcomes by leveraging the random allocation of genotypes, mimicking a randomized controlled trial (Sanderson et al., 2022; Ference et al., 2021). By integrating MR with expression quantitative trait loci (eQTL) data which are genetic variants associated with gene expression levels, it is possible to assess the causal effect of lifelong variation in the expression of a specific druggable gene on disease risk.

In this study, we performed a systematic, druggable genome-wide MR analysis by integrating the largest available IBS genome-wide association study (GWAS) summary statistics with comprehensive blood eQTL data. Our primary objective was to identify and prioritize novel, genetically validated drug targets for IBS. By further employing colocalization analysis to ensure the robustness of our findings, we identified EP300 and P2RY14 as promising candidate targets. Furthermore, by drug effect prediction and molecular docking, we inferred the pharmacological activities of candidate pharmaceutic agents targeting EP300 for IBS treatment. This study provides a valuable genetic foundation to guide future drug development and repurposing efforts, potentially accelerating the path toward more effective therapies for IBS.

## 2 Materials and methods

### 2.1 Druggable genes list

Druggable genes are defined as genes encoding proteins that can be targeted by drugs. The proteins encoded by these genes can specifically bind drug molecules to mediate their biological effects, making them potential targets in drug development (Hopkins and Groom, 2002). Drug-Gene Interaction Database (DGIdb, https://www.dgidb.org/) (Freshour et al., 2020) integrates comprehensive drug-gene interaction data, enabling efficient identification of potential druggable targets to support both biomedical research and clinical decision-making (Supplementary Table S1). Finan et al. integrated GWAS data with the druggable genome to systematically assess the utility of genetic associations in drug target discovery and therapeutic repurposing, revealing substantial cross-disease target opportunities and providing a scalable framework for target identification (Supplementary Table S2) (Finan et al., 2017). We compiled a list of druggable genes by integrating data from the DGIdb database and the review by Finan et al., excluding pseudogenes (Supplementary Table S3).

### 2.2 eQTL data

eQTLGen Consortium (https://eqtlgen.org/) (Võsa et al., 2021) is a comprehensive resource that integrates multiple datasets to elucidate the genetic basis of gene expression regulation. We obtained complete cis-eQTL data and allele frequency statistics from the eQTLGen Consortium, comprising 31,684 blood samples and 19,960 genes, all from individuals of European ancestry (Supplementary Table S4).

### 2.3 IBS data sources

Genome-wide association study (GWAS) data for the IBS cohort were obtained from the GWAS Catalog. The IBS GWAS (Study ID: GCST90016564) included 53,400 cases and 433,201 controls, all of European ancestry. Summary statistics and cohort details are available in the original publication (Supplementary Table S4) (Eijsbouts et al., 2021).

### 2.4 Mendelian randomization analysis

In this study, eQTLs were used as exposure variables. Instrumental variables (IVs) were selected as SNPs significantly associated with gene expression (false discovery rate [FDR] < 0.05) and located within ±100 kb of the transcription start site (TSS) of each target gene. To ensure SNP independence, linkage disequilibrium clumping was performed using genotype data from the European (EUR) population of the 1000 Genomes Project, with an *r* ^2^ threshold of 0.1 (Auton et al., 2015). Subsequently, phenotype annotation of the selected SNPs was performed using the “FastTraitR” (version 1.0.0) (Sollis et al., 2022) to identify and exclude variants associated with potential confounders of IBS, including abdominal obesity, gastrointestinal infection, somatic symptoms, copper and zinc levels, gluten-free diet, and asthma (Supplementary Table S5) (Zhu et al., 2024; Chen et al., 2024). Finally, independent SNPs with no evidence of significant pleiotropy were retained and used as instrumental variables in a two-sample Mendelian randomization (MR) analysis, leveraging GWAS summary statistics for IBS to assess the potential causal effect of gene expression on IBS risk.

In the primary analysis, for genes with a single valid instrumental variable, the causal effect was estimated using the Wald ratio method, defined as the ratio of the SNP’s effect on the outcome to its effect on the exposure. For genes with multiple independent instrumental variables, the inverse-variance weighted (IVW) method was used as the primary approach to estimate the overall causal effect, with MR-Egger regression and weighted median methods applied for sensitivity analysis to assess robustness and potential pleiotropy (Burgess et al., 2023). Cochran’s Q test was used to assess heterogeneity among the causal effect estimates of individual SNPs. A P value <0.05 was considered statistically significant and suggestive of heterogeneity (Greco et al., 2015). Additionally, the intercept term from MR-Egger regression was used to test for the presence of horizontal pleiotropy (Bowden et al., 2015). To control for the risk of false positives due to multiple testing, false discovery rate (FDR) correction was applied at the gene level. An FDR threshold of <0.05 was considered statistically significant (Duan et al., 2024). To assess potential reverse causality and evaluate the robustness of the assumed causal direction, we performed Steiger’s directionality test. This test determines whether the genetic instruments explain more variance in the exposure than in the outcome, thereby supporting the directionality of the causal effect from exposure to outcome. MR analyses were conducted using the “TwoSampleMR” package (version 0.6.6) (Hemani et al., 2018) in R version 4.4.1.

### 2.5 Colocalization analysis

For genes that reached statistical significance in cohorts, we conducted colocalization analysis using the “coloc” package (version 5.2.3) (Zuber et al., 2022) to assess whether the association signals for IBS risk and gene expression (eQTLs) shared a common causal variant. Since a single SNP may reside within regulatory regions shared by multiple genes, its association with disease risk could be confounded by expression changes in more than one gene. Therefore, colocalization analysis was performed to determine whether a shared causal genetic variant underlies both genetic susceptibility to IBS and the expression levels of specific genes.

Specifically, for genes found to be significant in the MR analysis, we conducted colocalization analysis using GWAS summary statistics for IBS and eQTL data within a ±100 kb window centered on the TSS of each gene. Prior probabilities were set as follows: P_1_ = 1 × 10^−4^ (prior probability of a SNP being associated with gene expression), P_2_ = 1 × 10^−4^ (prior probability of a SNP being associated with IBS risk), and P_12_ = 1 × 10^−5^ (prior probability of a shared causal variant for both traits).

Colocalization analysis yielded posterior probabilities for five mutually exclusive hypotheses, PPH0: No association with either trait; PPH1: Association with gene expression only; PPH2: Association with IBS risk only; PPH3: Associations with both traits, but driven by distinct causal variants; PPH4: A single shared causal variant underlies both gene expression and IBS risk. When PPH4 ≥ 0.8, strong evidence of colocalization was considered to exist between the two signals, supporting a shared genetic basis for expression changes in the gene and IBS risk.

### 2.6 PheWAS analysis

To evaluate the safety of potential drug targets and predict the risk of adverse reactions that may arise from targeted interventions, we conducted a phenome-wide association analysis based on a high-quality, predominantly unrelated whole-genome sequencing subset of approximately 500,000 participants released by the UK Biobank. This analysis systematically assessed the associations between protein-coding variants and approximately 13,000 binary traits as well as 5,000 continuous traits.

### 2.7 Genetic regulation and tissue expression characteristics analysis of target genes

To minimize the potential influence of tissue specificity on the results, this study systematically validated the eQTLs of the identified target genes through a series of colocalization analyses based on the GTEx database (https://www.gtexportal.org/home/). Furthermore, we utilized The Human Protein Atlas database (https://www.proteinatlas.org) to further evaluate the mRNA and protein expression profiles of relevant genes across different tissues, thereby clarifying their expression characteristics in the target tissues.

### 2.8 Candidate drug prediction

The Drug Signatures Database (DSigDB, https://dsigdb.tanlab.org/) (Yoo et al., 2015) contains 22,527 gene sets, encompassing 17,389 unique compounds and covering 19,531 genes. This comprehensive resource provides robust support for the systematic identification of potential druggable targets. Based on key target genes identified through Mendelian randomization and colocalization analyses, we performed drug-gene matching using the DSigDB. This approach was used to identify candidate drugs significantly associated with these targets, predict their pharmacological activities, and explore their potential for drug repurposing (Uhlén et al., 2015).

### 2.9 Molecular docking

To evaluate the binding affinity and molecular interaction profiles between candidate drugs and their target proteins, we performed molecular docking analysis. This provides structural insights into their potential mechanisms of action and supports assessment of target druggability. The three-dimensional structure of the target protein was retrieved from the Protein Data Bank (PDB, http://www.rcsb.org/), while the three-dimensional structures of small-molecule ligands were obtained from the PubChem Compound Database (https://pubchem.ncbi.nlm.nih.gov/) in SDF format (Kim et al., 2024). All protein structures were prepared by removing crystallization water molecules and adding hydrogen atoms, while ligand structures were energy-minimized using OpenBabel 3.1.1. The active binding sites were predicted using proteins. plus (https://proteins.plus/), an online platform for protein structure analysis and binding site prediction. These predicted sites were used to define the docking regions for subsequent molecular docking analyses. Molecular docking was conducted using AutoDock 4.2.6 (http://autodock.scripps.edu/) (Morris et al., 2009), and the docking results were evaluated based on binding free energy. A binding free energy lower than −5.0 kcal/mol is typically considered indicative of potential binding, while values below −7.0 kcal/mol suggest stronger binding activity. The final docking conformations were visualized using PyMol 3.1.0 (https://www.pymol.org/) to identify key molecular interactions, such as hydrogen bonds and hydrophobic interactions.

### 2.10 Clinical sample collection

To validate the expression of EP300 in clinical samples, we retrospectively enrolled 10 patients diagnosed with IBS at Guangzhou Women and Children’s Medical Center between 2020 and 2025. The inclusion criteria for the IBS group were children, adolescents, or young adults aged with a clinical diagnosis of IBS and no organic gastrointestinal diseases, systemic inflammatory conditions, or significant comorbidities. Concurrently, 10 healthy controls matched for age and sex were randomly selected from the hospital’s health examination center. Detailed clinical baseline characteristics of all participants are summarized in Supplementary Table S9.

### 2.11 RNA extraction and reverse transcription quantitative PCR (RT-qPCR)

Whole blood samples were collected in EDTA tubes. After red blood cell lysis and centrifugation, total RNA was extracted using the TRIZOL method (Invitrogen, 15596018CN). Reverse transcription was performed to obtain cDNA using the HiScript III All-in-one RT SuperMix (Vazyme, R333-01). Quantitative real-time PCR (qPCR) was conducted on the QuantStudio 6 Flex (ABI) platform using SYBR Green qPCR Master Mix (EZB, A0012-R2), with β-actin as the internal control. The primer sequences used were: β-actin (Forward: 5′-GCA​CAG​AGC​CTC​GCC​TTT-3′, Reverse: 5′-TAT​CAT​CAT​CCA​TGG​TGA​GCT​GG-3′) and EP300 (Forward: 5′-TCT​GCA​AAC​AAT​CGA​GCG​GA-3′, Reverse: 5′-GAA​ACT​GGA​ACC​ATG​CCT​GC-3′). And all the procedures were performed according to the manufacturers’ manuals. Relative expression levels were calculated using the 2^−ΔΔCT^ method, and the statistical difference between groups was evaluated using the Wilcoxon rank-sum test.

### 2.12 Processing of bulk RNA-sequencing datasets

To robustly evaluate EP300 expression and its downstream targets in IBS, we obtained raw count data from two independent RNA-sequencing (RNA-seq) datasets, GSE166869 and GSE168759, via the Gene Expression Omnibus (GEO) database. Raw counts were normalized to Counts Per Million (CPM). To eliminate technical variations between the two studies, we applied the ComBat function from the sva R package to the log-transformed CPM matrix for batch effect correction. Principal Component Analysis (PCA) was performed to visualize sample distribution before and after batch correction. The final high-quality integrated dataset comprised expression profiles for 15,439 genes across 84 samples, consisting of 53 IBS patients and 31 healthy controls.

### 2.13 Gene regulatory network inference

We inferred the EP300-centered gene regulatory network based on the integrated IBS RNA-seq dataset using the arboreto Python package. Specifically, the GRNBoost2 algorithm was employed to infer a regulatory network among the known transcription factors and potential target genes through a gradient-boosting machine approach. Subsequently, to ensure the reliability of the predicted interactions, we utilized the pySCENIC workflow with the cisTarget database to prune the network. Only targets possessing significant enrichment of EP300-associated motifs in their cis-regulatory regions were retained, resulting in a high-confidence EP300 regulon.

### 2.14 Functional enrichment and regulon activity analysis

To assess the statistical significance of the overlap between the EP300 regulon targets and IBS-upregulated genes, a hypergeometric distribution test was performed using the phyper function in R. Gene Ontology (GO) biological process and Kyoto Encyclopedia of Genes and Genomes (KEGG) pathway enrichment analyses for the overlapping genes were conducted using the clusterProfiler R package, with a significance threshold of P < 0.05. For the bulk RNA-seq data, the overall activity score of the EP300 regulon for each sample was calculated using the single-sample Gene Set Enrichment Analysis (ssGSEA) algorithm.

### 2.15 Single-cell transcriptome profiling

To explore the cell-type specificity of the EP300 regulon, we utilized the full single-cell RNA-sequencing dataset from the Gut Cell Atlas (https://www.gutcellatlas.org/), containing approximately 428,000 intestinal cells. We filtered the dataset to include only cells from adult and pediatric donors, excluding fetal samples. Cell type annotations (major and detailed levels) were adopted from the original dataset. The activity of the IBS-associated EP300 regulon in individual cells was scored using the AUCell algorithm based on the log-normalized expression matrix. To quantitatively and statistically determine the enrichment of the regulon in specific cell lineages, the Expression-Weighted Cell-type Enrichment (EWCE) method was employed.

## 3 Results

### 3.1 Instruments for druggable genes

By integrating data from the DGIDB database and the review by Finan et al., and excluding pseudogenes, a total of 5,642 druggable genes were obtained (Supplementary Table S6). Subsequently, these genes underwent matching for cis-eQTLs, followed by further linkage disequilibrium analysis and exclusion of confounding factors. Ultimately, 26,101 eligible single nucleotide polymorphisms (SNPs) were selected as instrumental variables.

### 3.2 Candidate druggable genes

**FIGURE 1:**
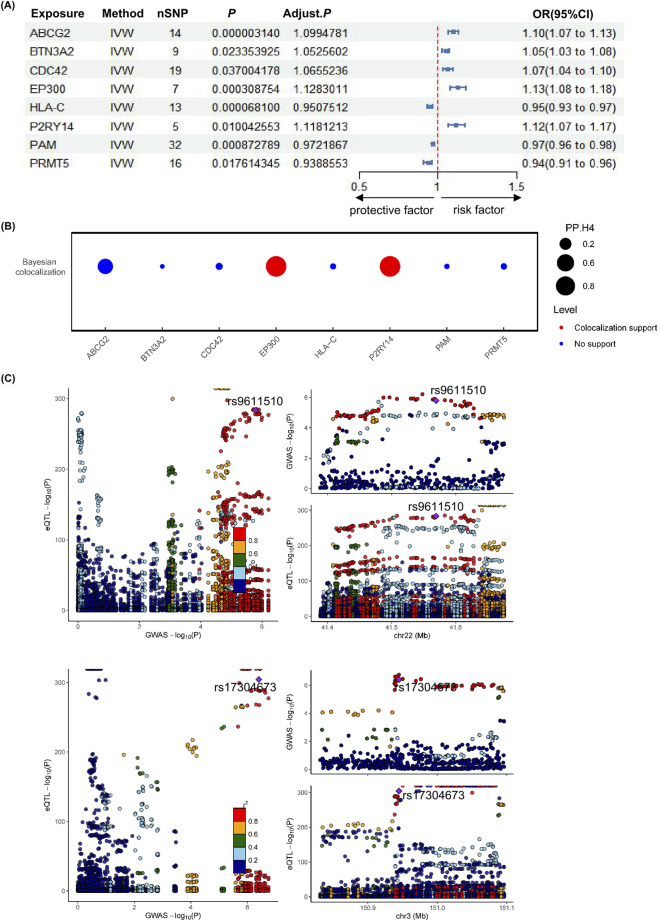
Mendelian randomization identification of genes and their co localization analysis. **(A)** Forest plot showing the MR associations between eQTL and IBS risk. **(B)** Bayesian colocalization analysis assessing the association between eQTL signals at specific gene/SNP loci and IBS. Red dots indicate significant colocalization evidence (posterior probability for hypothesis 4, PP.H4 > 0.8). **(C)** Bayesian colocalization of GWAS and eQTL signals. The genomic regions of *EP300* and *P2RY14* reveal strong colocalization signals (PP.H4 > 0.8).

Our study identified eight cis-eQTL genes with potential causal associations with IBS (FDR <0.05) (Supplementary Table S7). Among them, *HLA-C* (OR: 0.951, 95% CI: 0.934-0.968, P = 0.07 × 10–3, *PAM* (OR: 0.972, 95% CI :0.962 -0.983, P = 0.87 × 10–3), and *PRMT5* (OR: 0.939, 95% CI: 0.914 -0.965, P = 1.76 × 10–2) were identified as protective targets, whereas *ABCG2* (OR:1.099, 95% CI: 1.066 -1.133, P = 0.03 × 10–4, *BTN3A2* (OR: 1.053, 95% CI: 1.029 -1.076, P = 2.34 × 10–2), *CDC42* (OR :1.066, 95% CI: 1.036 -1.096, P = 3.70 × 10–2), *EP300* (OR: 1.128,95% CI: 1.079 -1.180, P = 0.31 × 10–3), and *P2RY14* (OR: 1.118,95% CI: 1.067-1.172, P = 1.00 × 10–2) were associated with increased risk of IBS (Figure 1A).

Sensitivity analyses were performed on the MR results to assess the robustness of the causal inferences. Cochran’s Q test showed no significant heterogeneity for any of the associations (P > 0.05), and MR-Egger regression indicated no evidence of substantial horizontal pleiotropy (P > 0.05). Furthermore, Steiger’s directionality test supported the direction of effect from gene expression to IBS risk, with no indication of reverse causality between the exposure and the outcome. These findings support a potential causal role of genetically predicted gene expression in IBS risk.

### 3.3 Colocalization analysis

Given that instrumental variable SNPs may be in linkage disequilibrium with independent causal variants, potentially leading to false positive associations in MR results, we further performed colocalization analysis to evaluate whether the exposure and outcome share the same causal SNP (Supplementary Table S8). Previous studies have demonstrated that integrating colocalization analysis significantly improves the success rate of MR in drug target discovery, thereby substantially enhancing the translational potential from genetic discoveries to therapeutic interventions. We performed colocalization analysis on eight key genes from MR screening (Figure 1B). Results showed two genes share the same IBS-risk causal variant (PP4 > 0.8) with statistically significant associations, indicating they may be potential pathogenic genes and therapeutic targets for IBS (Figure 1C).

### 3.4 Phenome-wide association analysis

**FIGURE 2:**
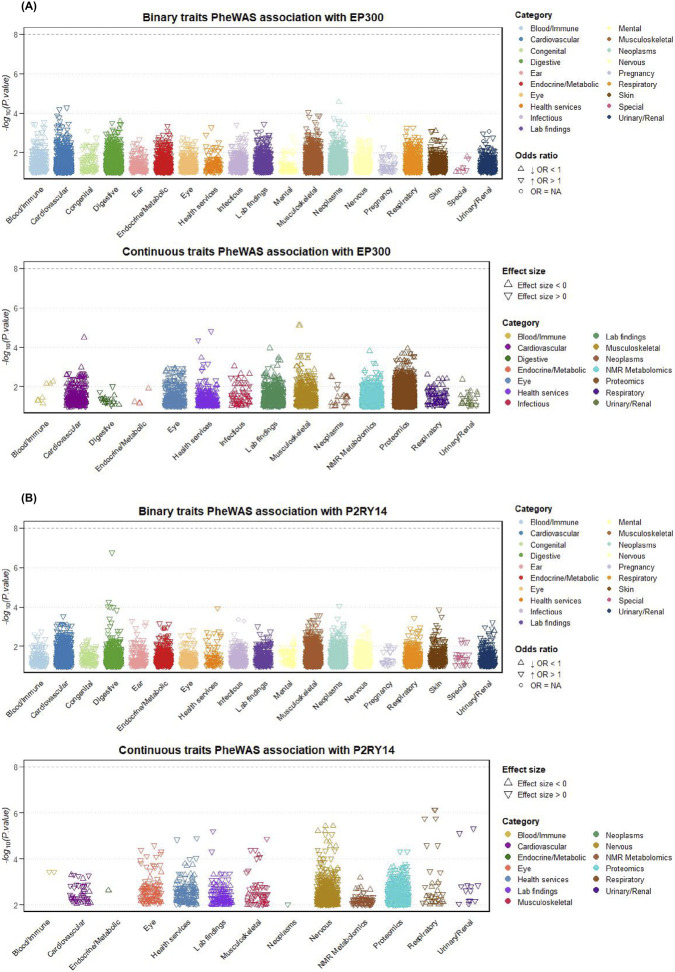
PheWAS analysis of *EP300* and *P2RY14*. **(A)** Binary and continuous trait PheWAS analysis of *EP300* gene. **(B)** Binary and continuous trait PheWAS analysis of *P2RY14* gene.

The results demonstrated that, at the genetic level, the candidate targets *EP300* and *P2RY14* did not show genome-wide significant associations (P < 10^–8^) with any other phenotypes (Figures 2A,B). This outcome implies that drug interventions targeting these two genes are likely to cause fewer cross-trait off-target effects, thereby indicating a relatively low potential for adverse side effects. It is worth noting that under the relatively relaxed nominal significance threshold (P < 1 × 10^−5^), the instrumental variables for EP300 and P2RY14 still show nominal associations with several non-IBS phenotypes, suggesting that this significance threshold may have an impact on the determination of the range of genetic associations.

### 3.5 Gene regulation and tissue expression profiling analysis

**FIGURE 3:**
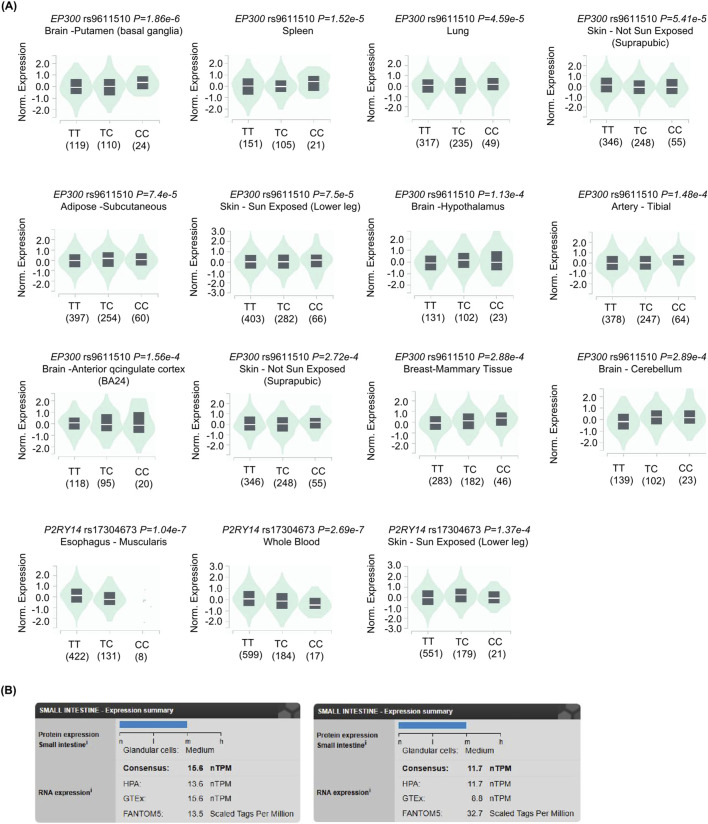
The regulatory effect of SNPs on genes and the expression of genes. **(A)** Distribution of expression levels for *EP300* and *P2RY14* across genotypes in individuals from the GTEx database. **(B)** Expression levels of EP300 and P2RY14 in human small intestine tissue.

To further investigate the regulatory role of genetic variation in gene expression and its potential association with IBS risk, we analyzed cis-eQTLs for the *EP300* and *P2RY14* genes using data from the GTEx database (Figure 3A). Our analysis revealed that the allele frequencies of relevant SNPs were significantly associated with the expression levels of both genes across multiple human tissues, suggesting their potential involvement in disease pathogenesis through the regulation of gene expression. Furthermore, we found that both EP300 and P2RY14 are expressed at moderate levels in the small intestine, indicating that they may exert biological functions locally within the gut (Figure 3B).

### 3.6 Targeted drug prediction and molecular docking

**FIGURE 4:**
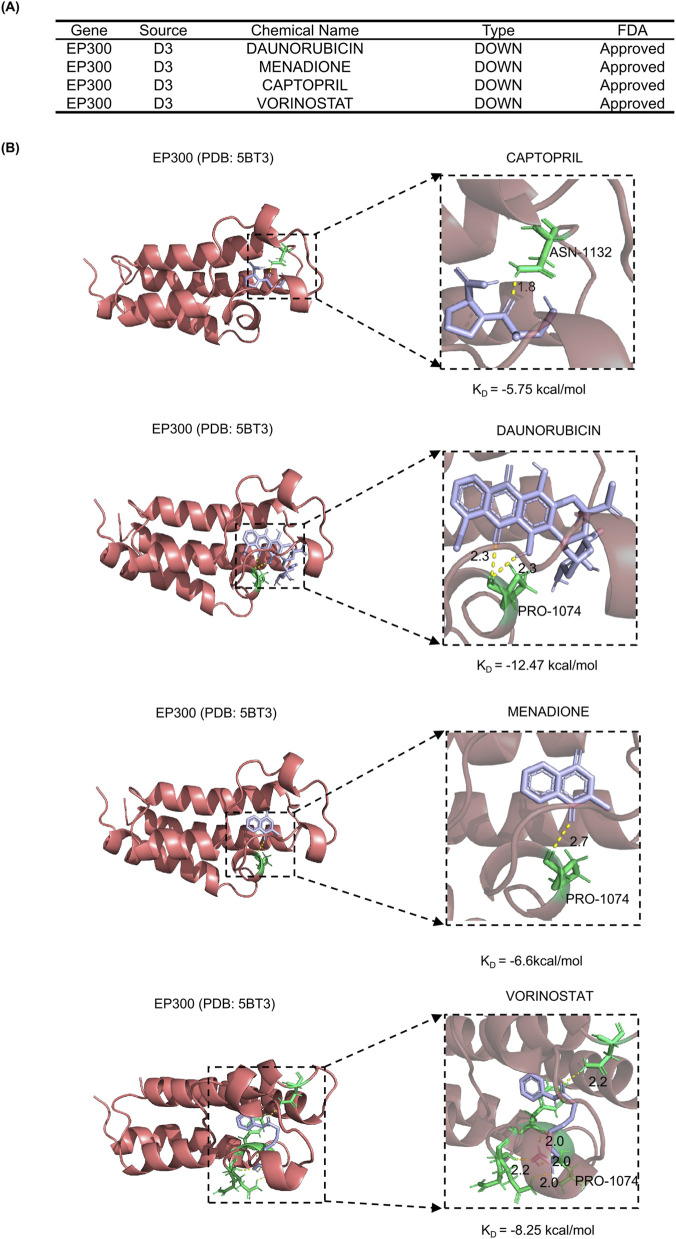
DSigDB prediction and molecular docking analysis. **(A)** Four small molecule compounds predicted to downregulate EP300 expression. **(B)** Molecular docking of EP300 protein and drugs.

We utilized the DSigDB to predict potential therapeutic compounds. The results indicated that four small-molecule compounds were predicted to have the potential to downregulate *EP300* gene expression. To further evaluate their ability to interact with the EP300 protein, molecular docking analysis was performed between these four candidate drugs and the EP300 protein (Figure 4A). The docking results suggested that DAUNORUBICIN exhibited the strongest binding affinity to the EP300 protein, with a binding energy of −12.47 kcal/mol. In addition, CAPTOPRIL, MENADIONE, and VORINOSTAT also showed favorable binding activity, suggesting that they may exert regulatory effects by directly binding to the EP300 protein (Figure 4B).

### 3.7 EP300 and its potential regulatory targets were upregulated in IBS

**FIGURE 5:**
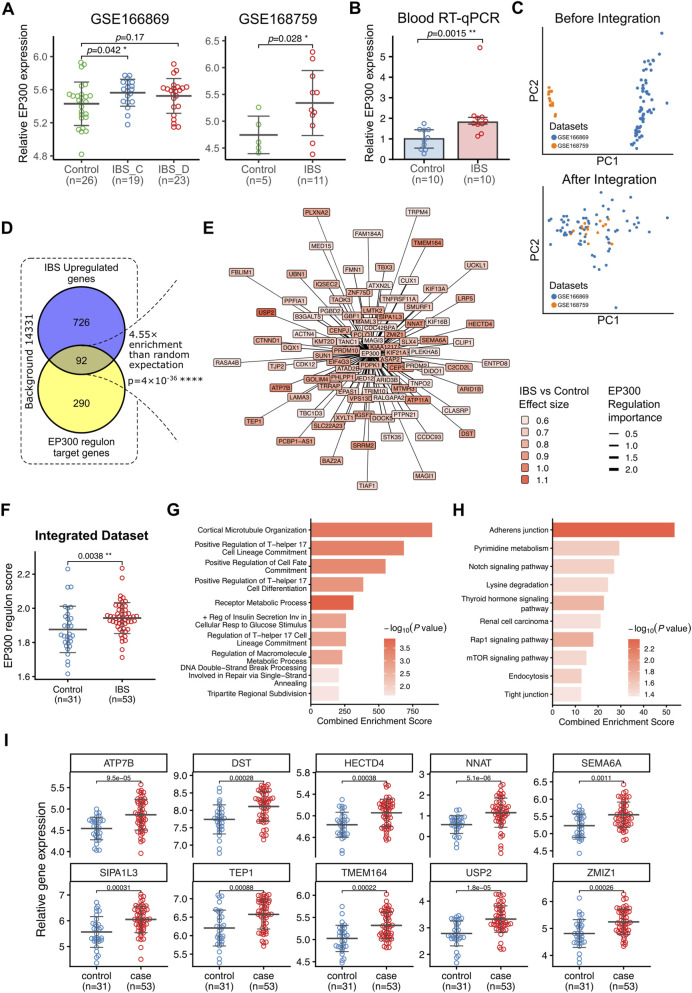
Association of EP300 and its transcriptional regulon with IBS. **(A)** Beeswarm diagrams illustrating the differential expression of EP300 between IBS and control samples in two public RNA-seq datasets: GSE166869 (left) and GSE168759 (right). Error bars indicate mean ± SD. Statistical significance of the results was determined using Student’s t-test. IBS-C: IBS with constipation; IBS-D: IBS with diarrhea. **(B)** Bar chart of EP300 expression levels in IBS and control blood samples determined by RT-qPCR. Bars represent median values of $2^{-\Delta \Delta CT}$ values of RT-qPCR experiment, and error bars represent 25% and 75% quantile. Statistical significance of the results was determined using Wilcoxon rank-sum test. **(C)** PCA scatter plots depicting sample distribution from GSE166869 and GSE168759 before (upper panel) and after (lower panel) batch effect correction using ComBat. **(D)** Venn diagram displaying the overlap between genes significantly upregulated in IBS patients compared to controls and EP300 regulon target genes identified using the GRNBoost2 + pySCENIC-cisTarget workflow (n = 382). **(E)** Network visualization of the 92 IBS-upregulated EP300 regulon target genes. Node colors represent the differential expression effect size (Cohen’s d) between IBS and controls, and edge widths represent regulation importance estimated by GRNBoost2. **(F)** Beeswarm diagram comparing EP300 regulon activity scores between IBS and control groups in the integrated dataset. Scores were calculated using ssGSEA based on all 382 EP300 regulon targets. **(G,H)** Bar charts showing the top enriched Gene Ontology (GO) biological processes **(G)** and KEGG pathways **(H)** for the 92 IBS-associated EP300 target genes. The x-axis represents the EnrichR Combined Enrichment Score. “+ Reg”: Positive regulation. **(I)** Combined beeswarm diagrams showcase the differential expression of top upregulated EP300 regulon target genes in IBS versus control groups.

To validate the expression characteristics of EP300 in IBS intestine, we employed two independent publicly-available RNA-sequencing datasets, GSE166869 (Grover et al., 2021) and GSE168759 (Edwinson et al., 2022). In the GSE166869 dataset, EP300 expression was significantly elevated in the IBS-C subgroup compared with healthy controls (Figure 5A, left). And this upregulation was further corroborated in the GSE168759 dataset (Figure 5A, right). To corroborate the *in silico* findings, we collected an independent clinical cohort consisting of blood samples from 10 IBS patients and 10 healthy controls with matched age and gender distribution (Supplementary Table S9), and the mRNA expression levels of EP300 were quantified using reverse transcription quantitative PCR (RT-qPCR). Consistently, EP300 expression was significantly elevated by approximately 1.8-fold in the blood of IBS patients compared to healthy controls (Figure 5B; Supplementary Table S9). These clinical results provide robust evidence supporting the upregulation of EP300 in IBS.

As EP300 is well-known as a transcriptional coactivator (Ma et al., 2021), we next sought to decipher the downstream regulatory network of EP300 to understand how it drives IBS pathology. We employed a stepwise analytic approach: First, we merged the two datasets (GSE166869 and GSE168759) and removed batch effects using the ComBat algorithm, as evidenced by the separation of dataset-specific clusters in PCA before correction and their cohesive integration afterwards (Figure 5C). Second, we utilized the GRNBoost2 algorithm (Moerman et al., 2019) to infer a regulatory network from the integrated dataset. Then, we applied the pySCENIC cisTarget workflow (Kumar et al., 2021) to prune this network based on transcription factor motif enrichment, retaining only high-confidence direct targets. This analysis identified a validated “EP300 regulon” consisting of 382 target genes. Motif analysis confirmed that the regulatory regions of these targets were significantly enriched for EP300-specific binding motifs (e.g., tfdimers__MD00332, NES >4.0), providing a physical basis for this regulation (Supplementary Table S10).

To specifically pinpoint the pathogenic component of this regulon, we mapped the 382 EP300 regulon targets to the 818 genes significantly upregulated in IBS identified from the integrated dataset, revealing a robust enrichment of the regulon within the disease signature, and this yielded a core set of 92 IBS-related EP300 regulon targets (Figure 5D). Network visualization highlights EP300 as a potential regulator driving the overexpression of these genes (Figure 5E). Consistently, the overall activity score of the EP300 regulon was significantly higher in IBS patients than in controls (Figure 5F), suggesting that EP300 hyperactivity may contribute to the transcriptomic alteration in IBS.

Functional enrichment analysis of the IBS-related EP300 regulon provided insights into the molecular mechanisms driven by EP300. GO analysis revealed enrichment in processes such as “cortical microtubule organization” and “positive regulation of cell fate commitment” (Figure 5G), while KEGG analysis highlighted the “Notch,” “Rap1,” and “mTOR” signaling pathways (Figure 5H). The upregulation of key targets involved in these pathways, such as *ATP7B*, *DST*, and *NNAT* (Figure 5I), suggests that EP300 may promote IBS pathogenesis by altering cell differentiation or renewal trajectories.

### 3.8 IBS-related EP300 regulatory targets were enriched in intestinal epithelium

**FIGURE 6:**
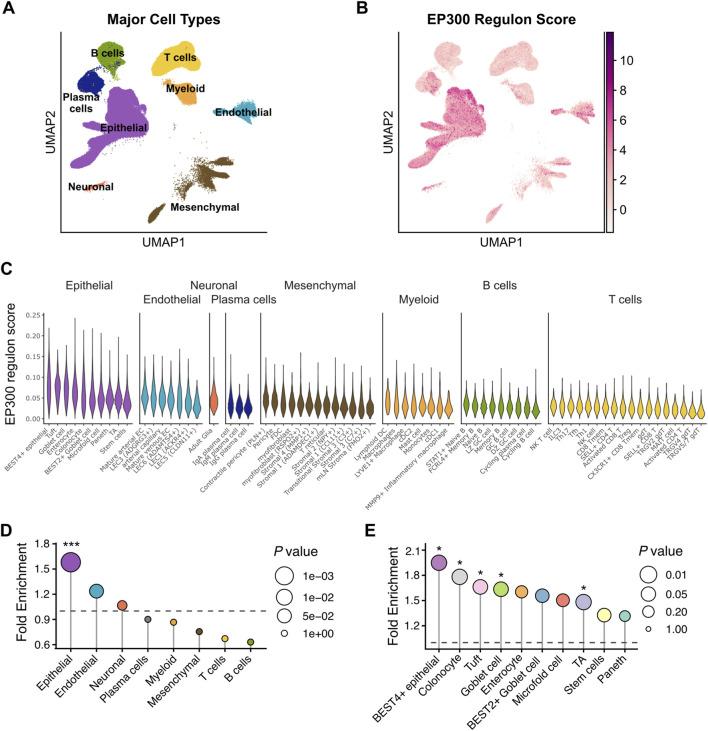
Profiling of the IBS-associated EP300 regulon in the intestinal single-cell transcriptome. **(A,B)** UMAP plots visualizing major cell types from the Human Gut Cell Atlas **(A)** and IBS-associated EP300 regulon scores among them **(B)**. Scores were calculated using AUCell based on the 92 IBS-upregulated target genes. **(C)** Violin plots displaying the distribution of IBS-associated EP300 regulon scores across different cell types. **(D,E)** Dot plots illustrating the cell-type enrichment of IBS-associated EP300 regulon target genes in major **(D)** and detailed **(E)** cell types using the EWCE analysis. The dashed line indicates a Fold Enrichment of one and significant enrichment is defined as Fold Enrichment >1 with P < 0.05.

To determine the major cellular source of this EP300-driven signature, we mapped the regulon activity onto the Gut Cell Atlas, a comprehensive single cell RNA-sequencing (scRNA-seq) dataset for human intestine (Elmentaite et al., 2021) (Figure 6A). Interestingly, the activity of the IBS-related EP300 regulon was highly enriched in the epithelial lineage, including absorptive enterocytes, secretory cells and progenitors, but low in immune subsets like B and T cells (Figures 6B,C). Consistently, Expression-Weighted Cell-type Enrichment (EWCE) (Skene and Grant, 2016) analysis confirmed that the EP300 regulon targets were only significantly enriched in the epithelial lineage, more specifically, BEST4^+^ epithelial cells, enterocytes, tuft cells and transit-amplifying epithelial cells (Figures 6D,E). These results suggested that the pathogenic effects of EP300 in IBS are more likely to be mediated through the disruption of intestinal epithelial homeostasis than a direct immune modulation.

## 4 Discussion

The primary objective of this study was to identify novel therapeutic targets for irritable bowel syndrome (IBS) by leveraging a systematic, druggable genome-wide Mendelian randomization (MR) approach. By integrating extensive genetic data from GWAS and eQTL studies, we provided robust evidence implicating EP300 and P2RY14 as promising candidate drug targets. Our findings indicate that genetically predicted higher expression of both genes is potentially causally associated with an increased risk of IBS. The subsequent phenome-wide association analysis further strengthened their candidacy by tentatively indicating a favorable safety profile that warrants further validation.

Our analysis highlighted the purinergic receptor P2RY14 as another candidate target. This G-protein coupled receptor is activated by extracellular UDP-sugars, which are often released during cellular stress or damage, and is highly expressed with highest expression in placenta, adipose tissue, stomach and intestine (Abbracchio et al., 2003; Zhang et al., 2023). Recent studies have demonstrated its pro-inflammatory role in the intestine. In preclinical disease models, P2Y14 has been implicated in gastrointestinal inflammation. For example, Liu et al. (2024) showed that P2RY14 expression is upregulated in colonic mucosa of ulcerative colitis patients, and genetic deletion or pharmacological blockade of P2Y14 ameliorates colitis via limiting epithelial necroptosis mediated by the cAMP/PKA/CREB → RIPK1 axis (Liu et al., 2024). Mechanistically, P2Y14 may amplify signals from damaged or stressed mucosa (as a DAMP receptor) to downstream MAPK or ERK pathways in eosinophils in large intestinal inflammation (Liu et al., 2026). Given that low-grade mucosal immune activation is implicated in the pathophysiology of IBS, genetic variation leading to higher P2RY14 expression could plausibly contribute to the development of IBS symptoms. Therefore, antagonizing the P2RY14 receptor may represent a promising therapeutic strategy for IBS.

Our study also identified EP300, which encodes the p300 protein, as a significant risk factor for IBS. As a central transcriptional co-activator and lysine acetyltransferase, p300 plays a crucial role in regulating chromatin structure and the expression of a wide range of genes involved in cell growth, differentiation, and inflammation (Attar and Kurdistani, 2017). Its dysregulation has been linked to various diseases, including cancer, fibrosis and inflammatory disorders (Attar and Kurdistani, 2017; Rubio et al., 2019; Yu et al., 2021). Notably, p300 is reported to be essential in the pathogenesis of inflammatory bowel disease (IBD) by altering H3K27ac modification near specific inflammation-related genes to trigger their expression (Yu et al., 2021). Therefore, we speculate that the function of EP300 in orchestrating inflammatory programs is also relevant to IBS, where low-grade mucosal inflammation is considered a key pathophysiological driver (Holtmann et al., 2016). Genetic variants influencing EP300 expression could therefore modulate the intestinal immune environment, providing a plausible biological mechanism for its association with IBS risk. These clues suggest that inhibition of the protein may potentially ameliorate IBS symptoms by dampening pro-inflammatory signaling pathways in the intestine.

The identification of EP300 as a druggable target opens avenues for drug repurposing. Prediction by DSigDB and our molecular docking analysis indicated that several existing compounds could bind to the EP300 protein, including the ACE inhibitor captopril and the synthetic vitamin K analog menadione. Interestingly, captopril, a well-tolerated antihypertensive drug, has demonstrated anti-inflammatory properties in preclinical colitis models (Salmenkari et al., 2026), making it a viable candidate for repurposing. Menadione has also shown promise by reducing inflammation in gastric and acute pancreatitis models via its modulating effect on the NF-кB signaling pathway (Lee et al., 2019; Amiti and Manickam, 2019). In contrast, chemotherapeutic agents like daunorubicin and vorinostat, despite showing binding potential, carry a high risk of severe toxicity and gastrointestinal side effects that would likely outweigh any potential benefits for a non-malignant condition like IBS. Therefore, safer compounds such as captopril warrant further investigation for their potential to modulate EP300 activity in the context of IBS. However, these computational predictions require further validation through cellular and animal preclinical experiments.

Our scRNA-seq profiling pinpointed the intestinal epithelium as the primary site of IBS-related EP300 regulon activity, a finding that resonates with the critical role of EP300 in maintaining intestinal epithelial homeostasis. Studies have established that p300 is indispensable for crypt cell proliferation and the maintenance of the intestinal stem cell niche, primarily through the Myb-p300 regulatory axis (Sampurno et al., 2013). Moreover, under pathological stress conditions, an EP300-dependent transcriptional program has been shown to coordinate the inflammatory activation of intestinal epithelial cells, while pharmacological inhibition of EP300 significantly protects epithelial integrity and restores intestinal ecology (Yin et al., 2023). This epithelial-related mechanism is particularly relevant to IBS, as compromised epithelial barrier function is well-documented pathophysiological hallmarks that link luminal perturbations to IBS (Hanning et al., 2021; Barbaro et al., 2024). Consequently, the epithelial-specific upregulation of the EP300 regulon identified in our study suggests that EP300 hyperactivity may contribute to IBS pathogenesis by disrupting the fine-tuned transcriptional control of epithelial differentiation and barrier maintenance, thereby leading to alteration in mucosal permeability and low-grade inflammation. This provides a potential mechanistic rationale for prioritizing EP300-targeted repurposed agents in future trials aimed at reinforcing the gut barrier.

This study has several notable strengths. To our knowledge, it is the first and most comprehensive druggable genome-wide MR analysis to identify potential therapeutic targets for IBS. By leveraging the largest available IBS GWAS dataset and employing a rigorous analytical framework that includes both MR and colocalization, we provide potentially robust, genetically validated evidence. Furthermore, the use of PheWAS to assess potential on-target side effects adds an important layer of translational relevance. Our findings not only reveal novel biological pathways potentially involved in IBS but also provide a data-driven foundation for prioritizing drug development and repurposing efforts.

Nevertheless, some limitations should be acknowledged. First, our analysis was based on individuals of European ancestry, and the findings may not be generalizable to other populations. Future studies in diverse ancestral groups are needed to confirm these results. Second, the eQTL data were derived from blood samples, which may not fully capture the tissue-specific gene expression patterns in the gastrointestinal tract. Although blood eQTLs often reflect systemic inflammatory processes relevant to IBS, validation with gut-specific eQTL data would strengthen our conclusions. Third, although qPCR and public datasets support the upregulation of EP300, the sample size of the local validation cohort in this study is relatively small, which may affect the precision of effect estimates, and residual confounding cannot be fully excluded. In addition, although the PheWAS did not reveal any signals for serious adverse reactions after strict correction, EP300 and P2RY14 show nominally significant associations with traits related to femur shaft bone and caudate nucleus (P < 1 × 10^−5^), suggesting that their biological effects may not be limited to the intestine. This not only reflects the shared mechanisms between IBS and comorbidities but also implies that future development of targeted therapies should be mindful of potential systemic effects. Finally, while MR analysis provides strong evidence for causality, the identified targets require further functional validation in preclinical models and ultimately in randomized controlled trials to confirm their therapeutic efficacy and safety for IBS treatment.

## Data Availability

The original contributions presented in the study are publicly available. This data can be found in the NHGRI-EBI GWAS Catalog under accession GCST90016564 and in the Gene Expression Omnibus (GEO) under accession numbers GSE166869 and GSE168759.
