# Supplementary material for: Multi-omics mendelian randomization integrating GWAS and eQTL data revealed potential drug target for irritable bowel syndrome
Source: Front Genet. 2026 May 19;17:1798264. doi: 10.3389/fgene.2026.1798264 (PMC13225775; doi:10.3389/fgene.2026.1798264)
Supplement: Supplementary file 3 [file Table9.docx]

| **Characteristic** | **Control (n=10)** | **IBS (n=10)** | ***P* value** |
| --- | --- | --- | --- |
| Gender (female\|male) | 3\|7 | 4\|6 | 1.00  (Fisher’s exact test) |
| Age (month, mean±SD) | 10.49±2.69 | 13.89±5.60 | 0.108  (Student’s t-test) |
| EP300 expression (median, (Q25, Q75)) | 1.00 (0.549, 1.44) | 1.80 (1.71, 2.05) | 0.00151  (Wilcoxon’s rank-sum test) |

Table S9. Characteristic of the subjects for validation of EP300 expression in blood. SD, standard deviation; Q25 & Q75, 25% and 75% quantile.

| **Motif** | **AUC** | **NES** | **Motif Similarity Qvalue** | **Orthologous Identity** | **Annotation** |
| --- | --- | --- | --- | --- | --- |
| 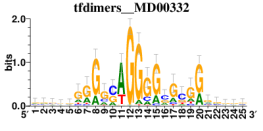 | 0.074209 | 4.123927 | 1.06E-07 | 1 | Gene is annotated for similar motif tfdimers__MD00537 ('M00033_forward_10_M01721_forward dimer: p300 / PUR1'; q-value = 1.06e-07) |
| 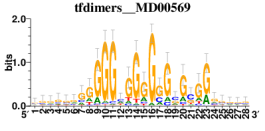 | 0.072868 | 3.986667 | 3.07E-09 | 1 | Motif similar to tfdimers__MD00537 ('M00033_forward_10_M01721_forward dimer: p300 / PUR1'; q-value = 3.07e-09) which is directly annotated |
| 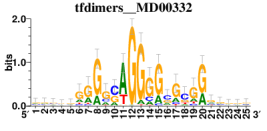 | 0.098437 | 3.958473 | 1.06E-07 | 1 | Gene is annotated for similar motif tfdimers__MD00537 ('M00033_forward_10_M01721_forward dimer: p300 / PUR1'; q-value = 1.06e-07) |
| 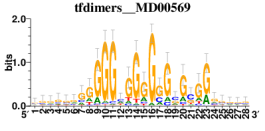 | 0.096304 | 3.823917 | 3.07E-09 | 1 | Motif similar to tfdimers__MD00537 ('M00033_forward_10_M01721_forward dimer: p300 / PUR1'; q-value = 3.07e-09) which is directly annotated |
| 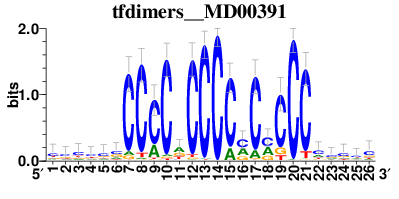 | 0.070671 | 3.761922 | 3.29E-06 | 1 | Gene is annotated for similar motif tfdimers__MD00537 ('M00033_forward_10_M01721_forward dimer: p300 / PUR1'; q-value = 3.29e-06) |

Table S10. Top 5 regulon motifs found by GRNBoost2 with pySCENIC cisTarget. AUC: Area-under-curve, NES: Normalized enrichment score.
